# Uveitis and Systemic Inflammatory Markers in Convalescent Phase of Ebola Virus Disease

**DOI:** 10.3201/eid2202.151416

**Published:** 2016-02

**Authors:** John R. Chancellor, Sriranjani P. Padmanabhan, Thomas C. Greenough, Richard Sacra, Richard T. Ellison, Lawrence C. Madoff, Rebecca J. Droms, David M. Hinkle, George K. Asdourian, Robert W. Finberg, Ute Stroher, Timothy M. Uyeki, Olga M. Cerón

**Affiliations:** University of Massachusetts Medical School, Worcester, Massachusetts, USA (J.R. Chancellor, S.P. Padmanablan, T.C. Greenough, R. Sacra, R.T. Ellison III, L.C. Madoff, R.J. Droms, D.M. Hinkle, G.K. Asdourian, R.W. Finberg, O.M. Cerón);; Massachusetts Department of Public Health, Boston, Massachusetts, USA (L.C. Madoff);; Centers for Disease Control and Prevention, Atlanta, Georgia, USA (U. Stroher, T.M. Uyeki)

**Keywords:** Ebola hemorrhagic fever, Ebola virus disease, Ebola, Ebola virus, viruses, uveitis, conjunctivitis, ocular manifestations, systemic inflammatory markers, immune privilege, convalescent phase, Liberia

## Abstract

We report a case of probable Zaire Ebola virus–related ophthalmologic complications in a physician from the United States who contracted Ebola virus disease in Liberia. Uveitis, immune activation, and nonspecific increase in antibody titers developed during convalescence. This case highlights immune phenomena that could complicate management of Ebola virus disease–related uveitis during convalescence.

## The Case-Patient

Fever developed in a physician providing health care in Liberia on August 29th, 2014 (day 0). The physician was positive for EBOV by reverse transcription PCR (RT-PCR) of plasma and was evacuated to the United States. Details of his acute phase clinical course and management were recently described (*1*).

The patient was given investigational drug TKM-100-802 siRNA LNP (Tekmira Pharmaceuticals, Burnaby, British Columbia, Canada). He also received convalescent-phase plasma from a survivor of EVD on day 9. During hospitalization, bilateral conjunctivitis (*1*) developed, but it resolved. He did not undergo formal ophthalmologic examination or report ocular symptoms and was discharged on day 26.

The patient came to the UMass Memorial Medical Center (Worcester, MA, USA) 37 days after onset of EVD with a 2-day history of nonproductive cough, low-grade fever, and generalized weakness. He was given azithromycin for suspected pneumonia. The patient reported irritation and redness of the left eye and was given topical polymyxin B sulfate/trimethoprim for presumed conjunctivitis. Blood cultures and nasal wash and swab specimens were negative for respiratory pathogens (Technical Appendix). RT-PCR result for a plasma specimen was negative for EBOV RNA.

The patient came to the UMass Memorial Eye Center on October 7, 2014 (day 40), with a 1-day history of painful vision loss, redness, and photophobia of the left eye. Results of review of other systems were negative. His medical history included treated latent tuberculosis and presumed acute Lyme disease treated in June 2014 with doxycycline.

**Figure 1:**
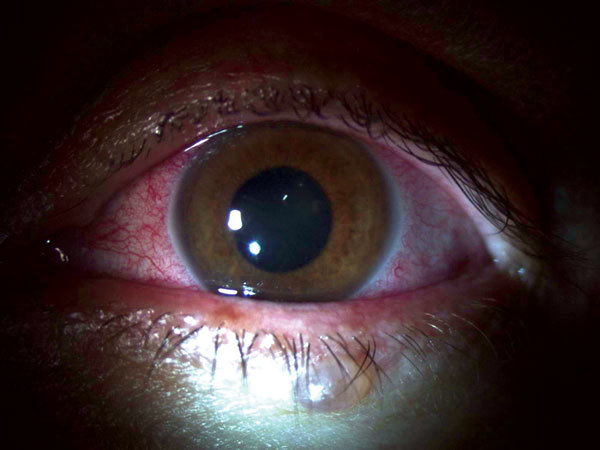
Slit lamp examination of the left eye of a physician from the United States who contracted Ebola virus disease in Liberia and had eye inflammation develop during convalescence. Image shows diffuse conjunctival injection, mild corneal edema with fine inferior keratic precipitates, fibrin reaction, and leukocytes in the anterior chamber without hypopyon. Used with permission of the patient.

He reported no history of ocular problems. Best corrected visual acuity was 20/25 in the right eye and 20/70 in the left eye. Intraocular pressures were 20 mm Hg in the right eye and 8 mm Hg in the left eye. Results of examination of the right eye were not remarkable. Slit lamp examination of the left eye showed conjunctival injection, mild corneal edema with fine inferior keratic precipitates, fibrin reaction, and leukocytes in the anterior chamber without hypopyon (Figure 1). The anterior vitreous humor was clear. The left fundus viewed by indirect ophthalmoscopy was hazy because of anterior segment findings but showed a grossly normal posterior segment. The patient was initially given topical 1% topical prednisolone acetate (every hour while awake) and 1% homatropine (2×/d). These drugs were gradually tapered over several weeks as he showed clinical improvement.

EBOV transmission was a concern because of reports of prolonged viral shedding on the ocular surface (*2*–*4*). After consent was obtained, the patient remained in home isolation pending results of conjunctival swab specimen testing. On day 42, one dry conjunctival swab specimen and 1 conjunctival swab specimen (in viral transport medium) from the inferior fornix of each eye were collected (Technical Appendix). Specimens were shipped to the Centers for Disease and Prevention (CDC; Atlanta, GA, USA), and all showed negative results by RT-PCR for EBOV RNA.

**Figure 2:**
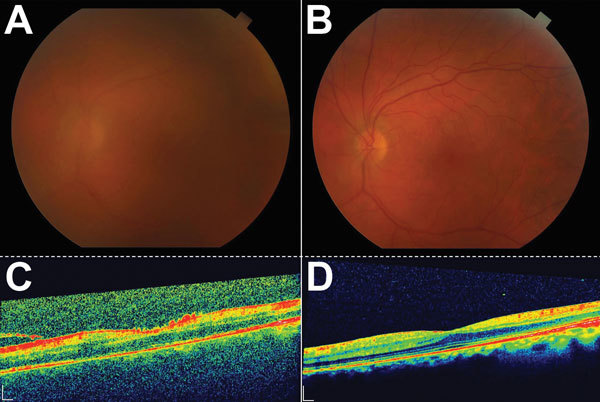
Color fundus and optical coherence tomography (OCT) images during active uveitis and after resolution for a physician from the United States who contracted Ebola virus disease in Liberia and had eye inflammation develop during convalescence. A) Color fundus image of the left eye showing a hazy view to the posterior pole during active uveitis (standardization of uveitis nomenclature classification grade 2–3). B) Color fundus image of the left eye showing a clear view to the posterior pole after resolution of uveitis. C) OCT of macula showing vitreous debris and small particles in a line of vitreous strands, consistent with inflammatory debris. D) OCT of macula showing resolution of vitreous and inflammatory debris. Scale bars indicate 200 μm.

On day 50, the patient had worsened best corrected visual acuity in the left eye (20/200) and increased floaters despite improved anterior chamber findings. Fundus examination demonstrated vitreous haze (standardization of uveitis nomenclature [*5*] classification grade 2–3 and classification grade 6 of Davis et al. [*6*]). No choroidal or retinal lesions were noted. Spectral domain optical coherence tomography (Heidelberg Engineering, Carlsbad, CA, USA) showed diffuse vitreous opacity and vitreous adhesions that appeared as small particles in a line of vitreous strands (Figure 2). In addition, spectral domain optical coherence tomography imaging showed cystoid macular edema and vitreous adhesions tethered to the optic disc.

Diagnostic studies for etiologies of uveitis were performed on day 54. Results were positive for human leukocyte antigen (HLA)–B27 haplotype. We found increased levels of IgM and IgG against cytomegalovirus, Epstein-Barr virus, and varicella zoster virus. Lyme disease screening with confirmatory Western blot showed IgM against *Borrelia burgdorferi* (IgG Western blot result was negative). The perinuclear–antineutrophil cytoplasmic antibody titer was 1:80, and the erythrocyte sedimentation rate (48 mm/h) was increased. Complete laboratory data are shown in the Technical Appendix. The patient showed persistence of EBOV RNA virus in semen during convalescence (CDC, unpub. data).

The patient was given prednisone (60 mg/d), and posterior segment inflammation had improved at follow-up 4 days later. Intraocular fluid sampling was considered for identifying EBOV RNA in aqueous or vitreous humors pending progress of the clinical course of the patient, but because his ocular inflammation responded well to medical therapy, this sampling was deferred.

Tapering of prednisone was initiated 1 week after treatment. Seven weeks (day 89) after initial presentation, left eye visual acuity increased to 20/25. As of March 2015, the patient was asymptomatic and his visual acuity was 20/20. The posterior segment was clear, and repeat spectral domain optical coherence tomography confirmed normal macular structure (Figure 2).

We implemented universal precautions during examinations and designated 1 examination room and a set of equipment for office visits. Personal protective equipment used is detailed in the Technical Appendix. Signs or symptoms of illness did not develop in any staff who cared for the patient.

## Conclusions

EVD evolved in December 2013 from a regional outbreak in West Africa to a major global health concern (*7*). As the number of survivors of Ebola increases, evaluation of ocular disease, particularly uveitis, will be a major component of patient management. Evaluation of non-EVD causes of uveitis in this patient showed notable results. First, he was positive for HLA-B27. This major histocompatibility class I allele has a well-recognized association with anterior uveitis (*8*). It is unclear whether this HLA status contributed to development of uveitis in the context of recent EVD. Second, there was evidence of global immune activation (increased erythrocyte sedimentation rate) and dysregulation of antibody production given the broad spectrum of positive serologic results, all of which fully returned to reference values on repeat testing after corticosteroid treatment (Technical Appendix).

Lymphocyte responses have been described in humans with EVD, and sustained responses have been noted up to 60 days after symptom onset (*9*). Prolonged presence of activated virus-specific lymphocytes might be caused by retained viral antigen despite undetected EBOV viral load. Antibody-producing plasmablasts levels increased during acute EVD, and measures of EBOV-specific responses suggested polyclonal expansion, including cells with other specificities (*9*). The pronounced increase in levels of serologic markers observed for this patient suggests that dysregulation of antibody production might contribute to immunopathogenesis and provides supporting evidence of a robust inflammatory response during EVD. Health care providers should be aware that the usual laboratory workup for uveitis might be confounding in the setting of recent EBOV infection.

We considered the possibility of an immune reaction secondary to recent treatment with TKM-100-802 siRNA LNP and convalescent-phase plasma from survivors of EVD. However, little data are available regarding these treatments and immune potentiation or uveitis. Larger trials would be necessary to evaluate these potential associations.

Similar to results of Varkey et al. (*10*), we did not detect EBOV RNA in conjunctival samples. However, the findings of viable EBOV persisting in aqueous humor during convalescence (*10*) and EBOV in semen in convalescent-phase samples (*2*,*11*) provides evidence that EBOV is harbored in immune-privileged organs. EBOV RNA was detected in semen specimens from our patient, and testing has yielded positive results (CDC, unpub. data). It remains unclear whether EVD-associated uveitis is caused by cytopathic effect of the virus or immune response, but early use of systemic corticosteroids appears to be beneficial, and sampling of intraocular fluid might not be necessary in patients who clinically improve with medical therapy.

**Technical Appendix.** Additional information on uveitis and systemic inflammatory markers in convalescent phase of Ebola virus disease.
