# Supplementary material for: Uveitis and Systemic Inflammatory Markers in Convalescent Phase of Ebola Virus Disease
Source: Emerg Infect Dis. 2016 Feb;22(2):295–7. doi: 10.3201/eid2202.151416 (PMC4734519; doi:10.3201/eid2202.151416)
Supplement: Supplementary file 1 — Technical Appendix. Additional information on uveitis and systemic inflammatory markers in convalescent phase of Ebola virus disease. [file 15-1416-Techapp-s1.pdf]

Article DOI: http://dx.doi.org/10.3201/eid2202.151416

Uveitis and Systemic Inflammatory Markers in Convalescent Phase of Ebola Virus Disease

Technical Appendix

Conjunctival Swab Specimen Collection during Ebola Virus Disease

1) Don (put on) personal protective equipment (PPE) in compliance with Centers for Disease Control and Prevention (CDC) recommendations during hospitalization for Ebola virus disease (*1*). When collecting specimens from patients who have recovered from Ebola virus disease, standard, contact, and droplet precautions should be followed. 2) Clean periorbital skin with sterile saline or sterile water and apply topical anesthetic as needed. 3) Pass a dry swab over the right lower conjunctival sac 5–6 times to obtain epithelial cells. 4) Immediately place swab into tube of viral transport medium. 5) Repeat the above process for the left eye. 6) Repeat for the right eye and left eye for a total of 2 swab specimens in viral transport medium for each eye. 7) Obtain 2 sets of swab specimens for each eye in a similar fashion for transport in dry containers (dry swab samples). 8) Doff (take off) PPE in compliance with CDC recommendations (*1*). 9) Contact the state public health department and refer to CDC guidance on collection, labeling, laboratory handling, transport, and submission of specimens potentially contaminated with Ebola virus (*2*).

Personal Protective Equipment

Standard, contact, and droplet precautions are recommended when evaluating patients who have survived Ebola virus disease and have ocular complications. Health care personnel must be trained and competent in correct use of recommended PPE, and a trained observer should monitor and confirm adherence to the proper use of PPE by others.

References

1. Centers for Disease Control and Prevention. Guidance on personal protective equipment to be used by healthcare workers during management of patients with Ebola virus disease in US hospitals, including procedures for putting on (donning) and removing (doffing) [cited 2015 Jul 1.] http://www.cdc.gov/vhf/ebola/healthcare-us/ppe/guidance.html

2. Centers for Disease Control and Prevention. Guidance for collection, transport and submission of specimens for Ebola virus testing [cited 2015 Jul 1]. http://www.cdc.gov/vhf/ebola/healthcare-us/laboratories/specimens.html

Technical Appendix Table 1. Laboratory results for possible etiology of uveitis in patient with Ebola virus disease, October 2014*

Test	Result	Comment	Reference value or range
ANCA screening with MPO/PR3, reflex to ANCA titer	P-ANCA positive	High	NA
Myeloperoxidase antibody titer	<1.0	Negative	<1.0: no antibody detected; ≥1.0: antibody detected
Proteinase 3 antibody titer	<1.0	Negative	<1.0: no antibody detected; ≥1.0: antibody detected
Complement C3c, mg/dL	85	Low	90–180
Complement C4c, mg/dL	15	Low	16–47
Complement C50, total, U/mL	41	Negative	31–60
CRP, mg/L	<1.0	Negative	<10
ESR, Westergren, mm/h	48	High	0–20
Rheumatoid factor, IU/mL	12	Negative	<14
Cyclic citrullinated peptide IgG titer, U	<1:16	Negative	<20: negative; 20–39: weak positive; 40–59: moderate positive; >59: strong positive
Cytomegalovirus IgG titer	3.98, positive	High	≤0.90: negative; 0.91–1.09: equivocal; ≥1.10: positive
Cytomegalovirus IgM titer	>4.0, positive	High	≤0.8: no antibody detected; 0.9–1.0: equivocal; ≥1.1: antibody detected
EBV IgG titer	>5.00, positive	High	≤0.90: negative; 0.91–1.09: equivocal; ≥1.10: positive
EBV IgM titer	4.28, positive	High	≤0.90: negative; 0.91–1.09: equivocal; ≥1.10: positive
EBV nuclear antigen antibody titer	>5.00, positive	High	≤0.90: negative; 0.91–1.09: equivocal; ≥1.10: positive
HSV 1 IgG/IgM titer	<0.90	Negative	≤0.90: negative; 0.91–1.09: equivocal; ≥1.10: positive
HSV 2 IgG/IgM titer	<0.90	Negative	≤0.90: negative; 0.91–1.09: equivocal; ≥1.10: positive
HIV-1 DNA, qualitative TMA	NR	NA	NA
Lyme disease antibody screening	2.64	Positive	≤0.90: negative; 0.91–1.09: equivocal; ≥1.10: positive
Lyme disease IgG	Negative	NA	NA
23-kD IgG band	Reactive	NA	NA
41-kD IgG band	Reactive	NA	NA
Lyme disease IgM	Positive	NA	NA
23-kD IgM band	Reactive	NA	NA
39-kD IgM band	Reactive	NA	NA
41-kD IgM band	Reactive	NA	NA
Toxoplasma IgG titer	≤0.90	Negative	≤0.90: negative; 0.91–1.09: equivocal; ≥1.10: positive
Toxoplasma IgM titer	Negative	NA	NA
Uric acid, mg/dL	3.7	Negative	2.5–8.0
VZV IgG titer	3.18, positive	High	≤0.90: negative; 0.91–1.09: equivocal; ≥1.10: positive
VZV IgM titer	2.40, positive	High	≤0.90: negative; 0.91–1.09: equivocal; ≥1.10: positive
Treponema pallidum, total FTA-ABS	NR	NA	NA

Test	Result	Comment	Reference value or range
Lysozyme, μg/mL	10.4	Negative	7.0–15.0
ACE, U/L	80	High	9–67
ANA screening by IFA	Negative	NA	NA
Malaria screening	Negative	NA	NA
CBC			
Leukocytes, × 10^3/mm^3	6.3	NA	4.3–10.3
Erythrocytes, × 10^6/mm^3	3.62	Low	4.40–6.00
Hemoglobin, g/dL	10.8	Low	4.40–6.00
Hematocrit, %	32.0	Low	42.0–52.0
MCV, fL	88.4	NA	82.0–101.0
MCH, pg	29.8	NA	27.0–34.0
MCHC, g/dL	33.7	NA	31.5–36.0
RDW, %	18.0	High	12.1–14.6
Platelet count, × 10^3/mm^3	312	NA	140–440
Mean platelet volume, fL	7.0	Low	7.6–11.6
Neutrophils, %	56.6	NA	43.0–72.0
Lymphocytes, %	39.8	NA	18.0–43.0
Monocytes, %	3.4	Low	4.0–12.0
Eosinophils, %	0.0	NA	NA
Basophils, %	0.2	NA	NA
Absolute no. neutrophils, × 10^3/mm^3	3.57	NA	NA
Absolute no. lymphocytes, × 10^3/mm^3	2.5	NA	NA
Absolute no. monocytes, × 10^3/mm^3	0.2	NA	NA
Absolute no. eosinophils, × 10^3/mm^3	0.0	NA	NA
Absolute no. basophils, × 10^3/mm^3	0.0	NA	NA
Erythrocyte morphology: anisocytosis	1+	NA	NA
Erythrocyte morphology: microcytosis	1+	NA	NA
Erythrocyte morphology: polychromasia	1+	NA	NA
Platelet morphology: large	Present	NA	NA
Platelet estimate	ADQ	NA	NA
PCR (respiratory pathogens)			
Adenovirus	ND	NA	NA
Bordetella pertussis	ND	NA	NA
Chlamydophila pneumoniae	ND	NA	NA
Coronavirus 229E	ND	NA	NA
Coronavirus HKU1	ND	NA	NA
Coronavirus NL63	ND	NA	NA
Coronavirus OC43	ND	NA	NA
Human metapneumovirus	ND	NA	NA
Human rhinovirus/enterovirus	ND	NA	NA
Influenza 2009 virus, H1 subtype	ND	NA	NA
Influenza A virus, H1 subtype	ND	NA	NA
Influenza A virus, H3 subtype	ND	NA	NA
Influenza B virus	ND	NA	NA
Mycoplasma pneumonia	ND	NA	NA
Parainfluenza virus 1	ND	NA	NA
Parainfluenza virus 2	ND	NA	NA
Parainfluenza virus 3	ND	NA	NA
Parainfluenza virus 4	ND	NA	NA
Respiratory syncytial virus	ND	NA	NA
Protein electrophoresis			
Protein, total, g/dL	7.3	NA	6.1–8.1
Interpretation	Normal pattern	NA	NA
β-globulins, g/dL	0.8	NA	0.8–1.4
α2-globulins, g/dL	0.7	NA	0.5–1.0
α1-globulins, g/dL	0.3	NA	0.1–0.3
Albumin g/dL, g/dL	4.1	NA	3.5–4.7
γ-globulins, g/dL	1.5	NA	0.6–1.6
BMP			
Sodium, mmol/L	139	NA	135–145
Potassium, mmol/L	4.2	NA	3.5–5.3
Chloride, mmol/L	101	NA	97–110
Carbon dioxide, mmol/L	25	NA	24–32
Anion gap, mg/dL	13	NA	5–15
Glucose, mg/dL	97	NA	70–99
Blood urea nitrogen, mg/dL	12	NA	7–23
Urine creatinine, mg/dL	1.10	NA	0.60–1.30
Glomerular filtration rate, mL/min	>60	NA	>60

Test	Result	Comment	Reference value or range
Calcium (ionized, whole blood) mg/dL	4.6	NA	4.6–5.3

*ANCA, antineutrophil cytoplasmic antibody; MPO/PR3, myeloperoxidase/proteinase 3; P-ANCA, perinuclear ANCA; NA, not applicable; CRP, C-reactive protein: ESR, erythrocyte sedimentation rate; EBV, Epstein-Barr virus; HSV, herpes simplex virus; TMA, transcription-mediated amplification; NR, not reactive; VZV, varicella zoster virus; FTA-ABS, fluorescent treponemal antibody absorption test; ACE, angiotensin-converting enzyme; ANA, antinuclear antibody; IFA, immunofluorescence assay; CBC, complete blood count; MCV, mean corpuscular volume; MCHC, mean corpuscular hemoglobin concentration; RDW, red blood cell distribution width; ADQ, adequate; ND, not detected; BMP, basic metabolic panel.

Technical Appendix Table 2. Repeat laboratory results for possible etiology of uveitis in patient with Ebola virus disease, April 2015*

Test	Result	Comment	Reference value or range
ANCA screening with MPO/PR3, reflex to ANCA titer	Negative	Negative	NA
Myeloperoxidase antibody titer	<1.0	Negative	<1.0: no antibody detected; ≥1.0: antibody detected
PR3 antibody titer	<1.0	Negative	1.0: no antibody detected; ≥1.0: antibody detected
CRP (January 2015), mg/L	<1.0	Negative	<10
ESR, Westergren, mm/h	11	Negative	0–20
Cytomegalovirus IgG titer	2.78, positive	High	≤0.90: negative; 0.91–1.09: equivocal; ≥1.10: positive
Cytomegalovirus IgM titer	0.7	Negative	≤0.8: no antibody detected; 0.9–1.0 equivocal; ≥1.1: antibody detected
EBV IgG titer	>5.00, positive	High	≤0.90: negative; 0.91–1.09: equivocal; ≥1.10: positive
EBV IgM titer	<0.90	Negative	≤0.90: negative; 0.91–1.09: equivocal; ≥1.10: positive
EBV nuclear antigen IgG titer	>5.00, positive	High	≤0.90: negative; 0.91–1.09: equivocal; ≥1.10: positive
Lyme disease antibody titer	<0.90	Negative	≤0.90: negative; 0.91–1.09: equivocal; ≥1.10: positive
VZV IgG titer	2.62, positive	High	≤0.90: negative; 0.91–1.09: equivocal; ≥1.10: positive
VZV IgM titer	<0.90	Negative	≤0.90: negative; 0.91–1.09: equivocal; ≥1.10: positive

*ANCA, antineutrophil cytoplasmic antibody; NA, not applicable; MPO/PR3, myeloperoxidase/proteinase 3; CRP, C-reactive protein: ESR, erythrocyte sedimentation rate; EBV, Epstein-Barr virus; VZV, varicella zoster virus.
